# Auditory stimuli and heart rate variability: the role of music in cardiovascular regulation

**DOI:** 10.3389/fcvm.2026.1841349

**Published:** 2026-05-20

**Authors:** Predrag Mitrovic, Aleksandra Paladin

**Affiliations:** 1 Cardiology Clinic, Clinical Center of Serbia, Faculty of Medicine, University of Belgrade; 2 Faculty of Contemporary Arts, Music Production Department, Radio Television of Serbia, Belgrade

**Keywords:** autonomic nervous system, cardiovascular disease, heart rate variability, music therapy, non-pharmacological intervention

## Abstract

Music is a universal human experience with measurable physiological effects. Among these, its influence on heart rate and autonomic regulation has attracted increasing scientific attention. This paper explores the mechanisms through which music affects cardiac function, including neural pathways, emotional processing, and autonomic nervous system modulation. Additionally, it reviews the impact of different musical genres and tempos on heart rate and heart rate variability (HRV), as well as potential clinical applications in cardiology and rehabilitation.

## Introduction

The relationship between auditory stimuli and physiological regulation has gained increasing attention in recent decades. Music, as a structured and emotionally engaging auditory stimulus, exerts measurable effects on cardiovascular parameters, particularly heart rate (HR) and heart rate variability (HRV). These parameters are widely recognized as indicators of autonomic nervous system (ANS) balance and cardiovascular health (1, 2).

Previous studies have demonstrated that music can influence HR both acutely and chronically, suggesting its potential as a non-pharmacological intervention (1, 3). However, variability in study design, musical characteristics, and individual responses has led to inconsistent findings (4, 5). This paper aims to synthesize current knowledge regarding the effects of music on HR and HRV, with emphasis on physiological mechanisms and clinical applications.

This manuscript represents a narrative review based on a structured literature search conducted using PubMed, Scopus, and Web of Science databases. Keywords included “music”, “heart rate”, “heart rate variability”, and “autonomic nervous system”. Studies were selected based on relevance to cardiovascular outcomes and autonomic regulation.

### Neurophysiological mechanisms

**Figure 1:**
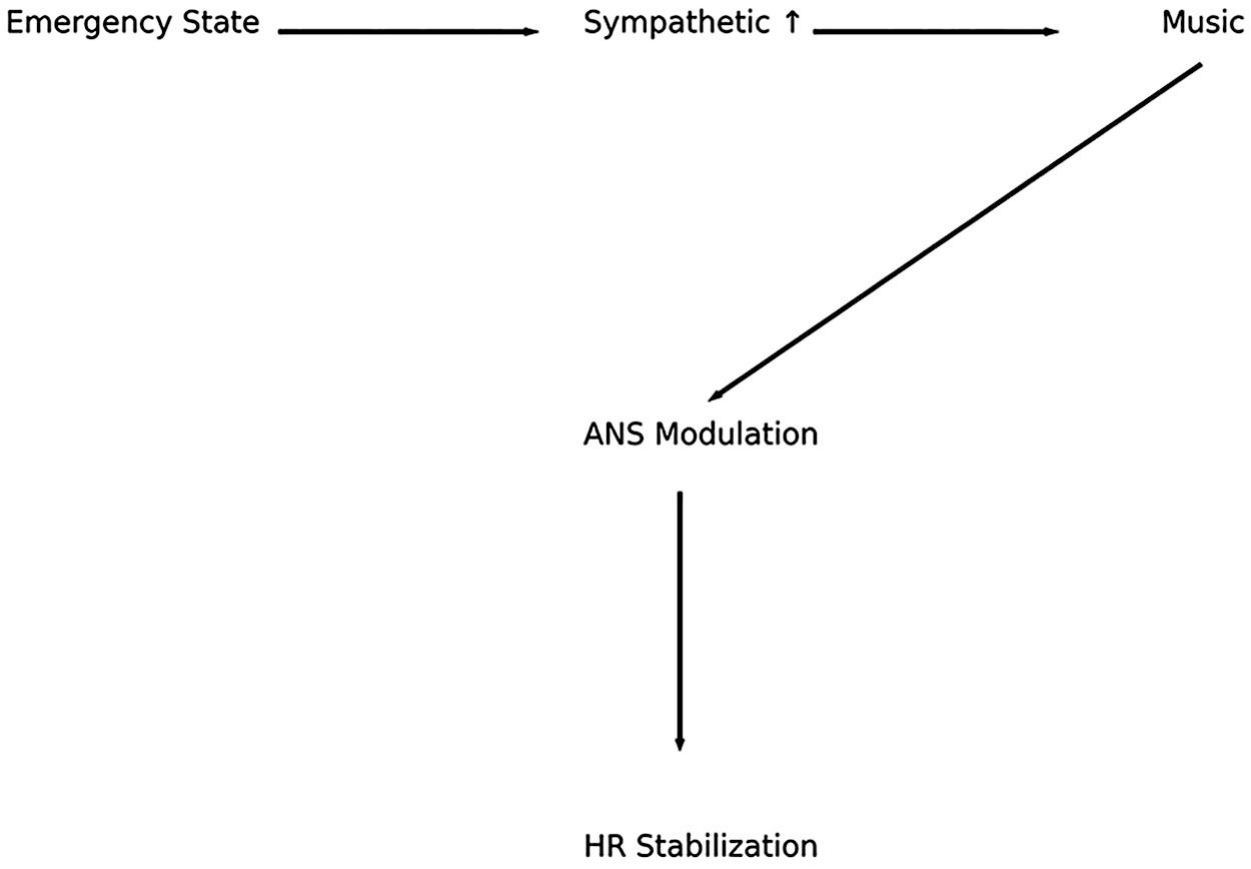
Neurocardiac integration of music processing. Schematic representation of the neurophysiological pathways involved in music perception and cardiovascular regulation. Auditory stimuli are processed in the auditory cortex and subsequently engage limbic structures, including the amygdala and hippocampus, which modulate hypothalamic output. This leads to regulation of the autonomic nervous system, influencing heart rate, vascular tone, and overall cardiovascular function.

The cardiovascular effects of music are mediated through interconnected neural pathways involving the auditory cortex, limbic system, and hypothalamic-autonomic axis (6, 7). Sound signals are initially processed in the primary auditory cortex, after which they engage emotional and cognitive centers, including the amygdala, hippocampus, and prefrontal cortex (Figure 1) (6).

The amygdala plays a central role in emotional evaluation of auditory stimuli and modulates autonomic output via projections to the hypothalamus (6, 8). The hypothalamus, in turn, regulates sympathetic and parasympathetic activity through brainstem centers, resulting in measurable changes in HR and vascular tone (8).

Neurochemical mechanisms further contribute to these effects. Dopaminergic pathways associated with reward processing are activated during pleasurable music listening, while endorphin release contributes to relaxation and stress reduction (7, 8). These processes collectively influence autonomic balance and cardiovascular responses.

### Effects of musical characteristics on heart rate

#### Tempo and rhythm

**Figure 2:**
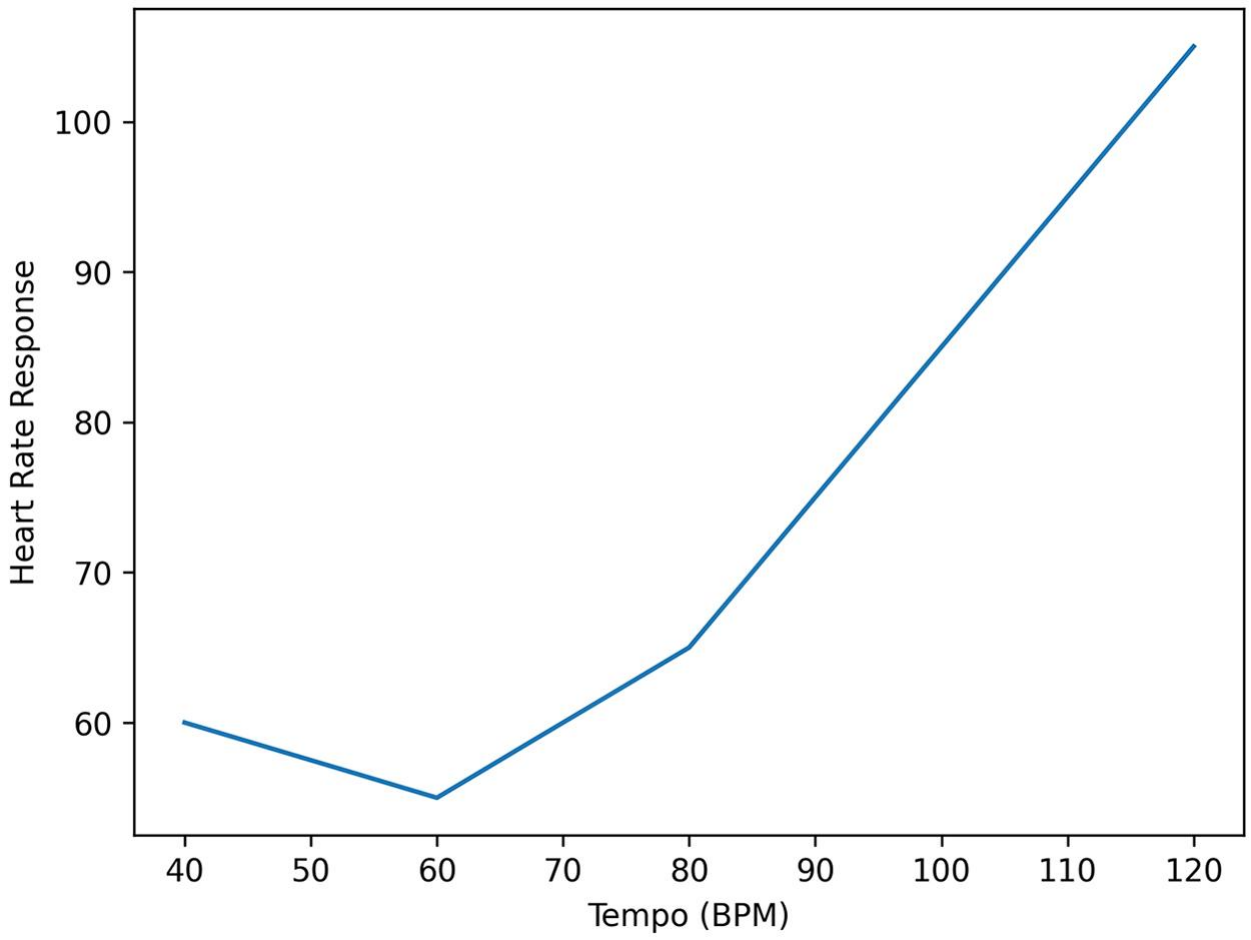
Relationship between music tempo and autonomic response. Conceptual illustration of the association between music tempo and autonomic regulation. Slow-tempo music (60–80 beats per minute) is associated with increased parasympathetic (vagal) activity and reduced heart rate, while fast-tempo music promotes sympathetic activation, leading to increased heart rate and physiological arousal.

Tempo is one of the most influential factors affecting cardiovascular response. Fast-tempo music (typically > 100–120 beats per minute) is associated with increased HR and sympathetic activation, whereas slow-tempo music (< 60–80 beats per minute) promotes parasympathetic dominance and HR reduction (Figure 2) (1, 9).

#### Dynamics and intensity

Higher sound intensity and abrupt dynamic changes may increase physiological arousal and HR. Conversely, softer and more stable sound patterns are associated with relaxation (10).

#### Harmony and predictability

Consonant and predictable harmonic structures tend to induce calming effects, whereas dissonant or unpredictable music may increase stress-related responses (10).

#### Individual preference

Subjective perception and familiarity significantly modulate physiological responses. Music perceived as pleasant enhances parasympathetic activity, while disliked music may provoke stress responses regardless of tempo (4, 5, 11).

### Heart rate variability and autonomic regulation

Heart rate variability (HRV) reflects the dynamic interplay between sympathetic and parasympathetic influences on the sinoatrial node and is widely used as a marker of cardiovascular adaptability (4, 9). Music has been shown to influence HRV in a direction consistent with its autonomic effects. Relaxing music increases high-frequency (HF) components of HRV, indicating enhanced vagal activity, while stimulating music may reduce HRV due to sympathetic dominance (9, 12). These findings suggest that music can serve as a tool for modulating autonomic balance in both healthy individuals and patients.

### Clinical applications in cardiovascular medicine

#### Cardiac rehabilitation

Music interventions have been associated with reduced HR, blood pressure, and anxiety in patients with cardiovascular disease (2, 13). In cardiac rehabilitation programs, music may improve adherence and psychological well-being (13).

#### Perioperative and intensive care settings

Music listening has been shown to reduce perioperative stress, lower HR, and decrease the need for sedatives in some patients (14, 15). In intensive care settings, music may improve patient comfort and reduce physiological stress markers (5, 14).

#### Chronic cardiovascular conditions

Music-based interventions may contribute to improved autonomic balance and reduced cardiovascular burden associated with chronic stress (4, 16).

### Music in emergency cardiology

#### Acute myocardial infarction (AMI)

**Figure 3:**
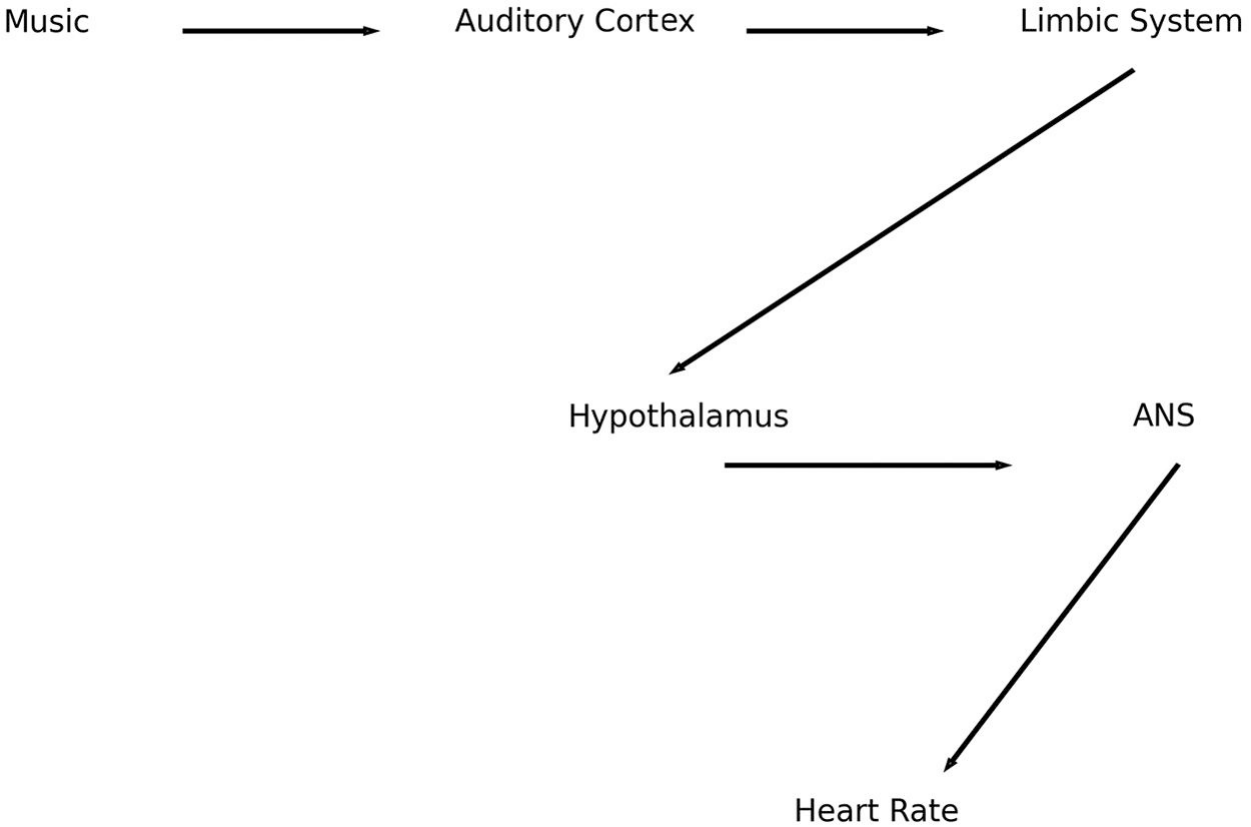
Clinical application model in emergency cardiology. Illustration of the potential role of music interventions in clinical settings. Acute cardiovascular conditions are associated with increased sympathetic activation and stress response. Music-based interventions may contribute to autonomic modulation, reduction of stress and anxiety, and stabilization of heart rate and other physiological parameters.

Patients with acute myocardial infarction exhibit marked sympathetic activation, which contributes to increased HR, myocardial oxygen demand, and arrhythmogenic potential. Music interventions may reduce HR and anxiety, potentially decreasing sympathetic drive and myocardial workload (Figure 3) (1, 2, 5).

#### Arrhythmias

Autonomic imbalance plays a key role in arrhythmogenesis. Music-induced parasympathetic activation may reduce the incidence of certain supraventricular arrhythmias and improve HRV parameters (4, 9). However, excessive auditory stimulation may have adverse effects in susceptible individuals.

#### Intensive care unit (ICU)

**Figure 4:**
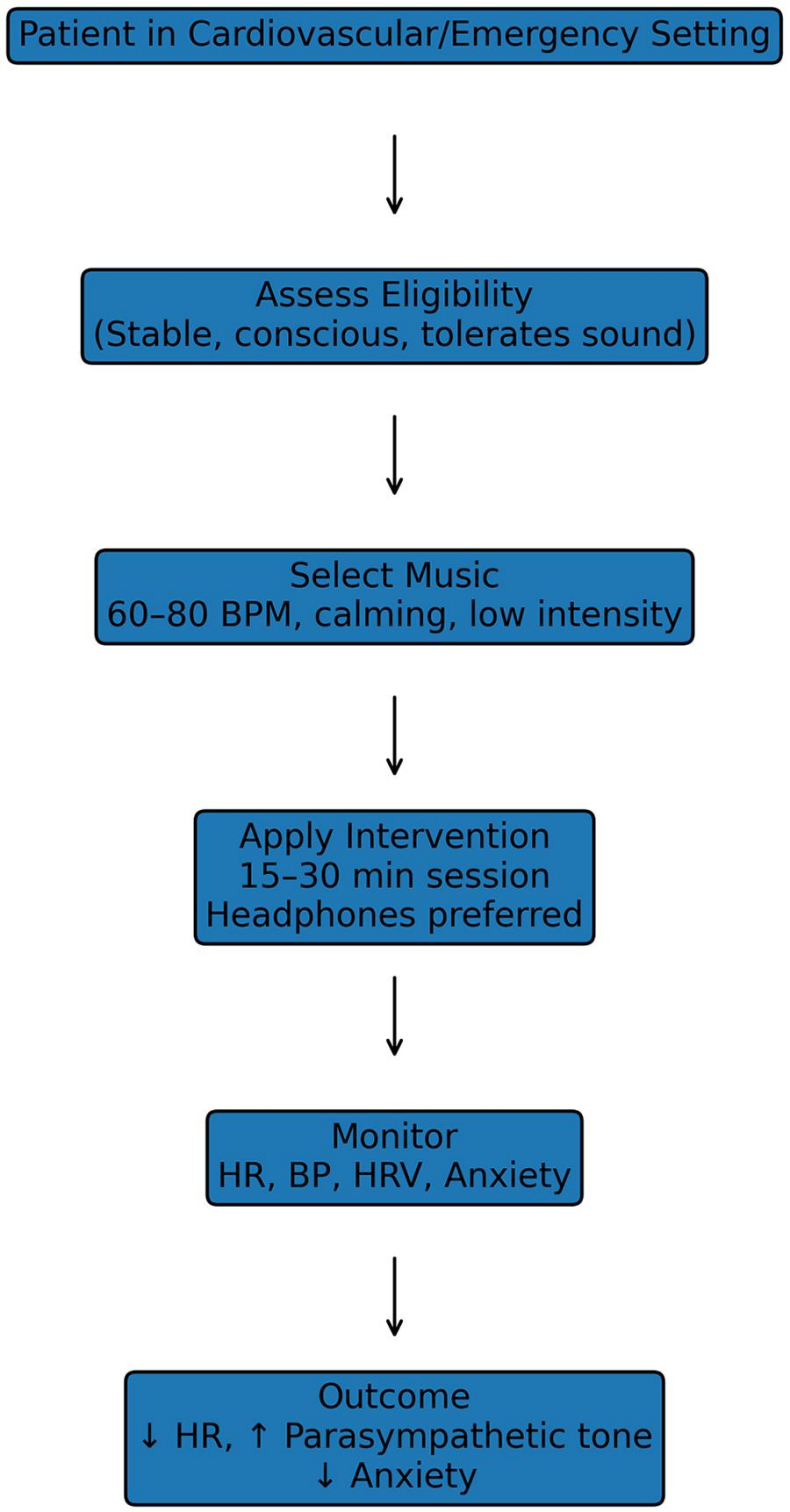
Proposed clinical flowchart for music-based intervention in cardiovascular and emergency care. Stepwise algorithm illustrating the implementation of music-based interventions in clinical practice. The flowchart includes patient assessment, eligibility criteria, selection of music parameters (tempo, rhythm, intensity), intervention delivery (duration and frequency), physiological monitoring (heart rate, blood pressure, heart rate variability), and evaluation of clinical outcomes. This framework is intended to support structured and reproducible application of music interventions across different clinical settings. The proposed model is based on current evidence and should be considered exploratory, requiring validation in future clinical studies.

Patients in intensive care units are exposed to continuous stress, noise, and sleep disruption. Music interventions in ICU settings have been associated with reduced HR, respiratory rate, and anxiety levels (Figure 4) (5, 14, 15). A reduction in heart rate is beneficial when it reflects improved autonomic balance, particularly reduced sympathetic overactivity, rather than pathological bradycardia.

#### Anxiety and pain in emergency settings

Pain and anxiety significantly increase sympathetic activity. Music may act as a non-pharmacological intervention that reduces perceived pain and lowers HR and blood pressure (15, 17).

## Discussion

The present review highlights the growing body of evidence supporting the influence of music on cardiovascular regulation, particularly through modulation of the autonomic nervous system (1, 4). However, despite promising findings, several critical limitations must be addressed before music can be fully integrated into evidence-based clinical practice.

First, heterogeneity across studies remains substantial. Variability in musical stimuli (tempo, genre, duration), patient populations, and outcome measures limits comparability and reproducibility (3, 4). Few studies employ standardized protocols, and even fewer incorporate objective autonomic biomarkers such as spectral HRV analysis or catecholamine levels (9, 12).

Second, individual variability represents a significant confounding factor. Emotional and cognitive responses to music are highly personalized, suggesting that uniform interventions may not yield consistent physiological outcomes (4, 11). This underscores the importance of individualized or adaptive music-based interventions.

Third, the majority of available data originates from controlled or non-acute settings. Evidence in emergency cardiology, particularly in high-risk populations such as acute myocardial infarction or unstable arrhythmias, remains limited (1, 2, 13). This represents a critical gap in the literature and an important direction for future research.

From a mechanistic perspective, the interaction between auditory processing, emotional regulation, and autonomic output suggests that music operates as a multisystem modulator rather than a simple sensory stimulus (6–8). Music processing involves coordinated activation of the auditory cortex, limbic structures, and hypothalamic centers, which collectively regulate autonomic output. Through these pathways, music influences both emotional states and physiological responses, including heart rate and vascular tone. This integrative effect may explain its potential to influence cardiovascular parameters beyond traditional behavioral interventions.

Future studies should prioritize: 1) standardization of music protocols (tempo ranges, duration, delivery method) (4), 2) use of objective physiological endpoints (HRV, neurohormonal markers) (9, 12), 3) randomized controlled trials in acute and emergency settings (13), 4) development of personalized music intervention models (4, 11). In this context, music may evolve from a complementary therapy into a structured component of integrative cardiovascular care.

Given the limited number of studies in emergency cardiology, the proposed applications should be considered exploratory and hypothesis-generating, requiring validation in future randomized controlled trials.

Emerging technologies, including artificial intelligence and wearable devices, may enable real-time adaptation of music interventions based on physiological parameters such as heart rate variability, paving the way for personalized and precision-based music therapy approaches.

### Evidence synthesis

Current evidence consistently demonstrates that music influences cardiovascular parameters through autonomic modulation (1, 2, 4). Across multiple studies, slow-tempo and relaxing music is associated with reductions in heart rate and increases in parasympathetic activity, as reflected by HRV indices (9, 12). Conversely, stimulating or high-tempo music tends to increase sympathetic activation (1, 9). Meta-analytic data suggest modest but statistically significant reductions in heart rate and blood pressure in patients exposed to music interventions, particularly in perioperative and rehabilitation settings (3, 13). However, effect sizes vary considerably depending on study design and intervention characteristics (3).

Importantly, the translation of these findings into acute care settings remains limited. While preliminary data indicate potential benefits in stress reduction and autonomic stabilization, high-quality randomized trials in emergency cardiology are lacking (13, 14) (Table 1).

**Table 1:** Summary of key studies investigating the effects of music interventions on cardiovascular and autonomic parameters.

Author	Year	Study Type	Population	Intervention	Outcome	Level of Evidence
Bernardi et al. (1)	2006	Experimental study	Healthy subjects	Music with varying tempo	Changes in HR, BP, respiration	II
Iwanaga et al. (9)	2005	Experimental study	Healthy adults	Repetitive music exposure	Modulation of HRV	II
Bradt et al. (13)	2013	Systematic review	Cardiac patients	Music interventions	Reduced HR, anxiety	I
Nilsson (15)	2008	Clinical study	Surgical patients	Music therapy	Reduced anxiety and HR	II
Thoma et al. (11)	2013	Randomized controlled trial	Healthy volunteers	Music vs. control condition	Reduced stress markers, HR	I
Knight and Rickard (18)	2001	Experimental study	Healthy adults	Relaxing music	Prevention of stress-induced HR increase	II
Pelletier (3)	2004	Meta-analysis	Mixed populations	Music exposure	Reduced stress and arousal	I
Loomba et al. (2)	2012	Clinical study	Cardiovascular patients	Music therapy	Reduction in BP and HR	II
White (19)	1999	Clinical study	ICU patients	Relaxing music	Improved autonomic balance	II
Raglio et al. (16)	2015	Clinical study	Neurological/cardiac patients	Music therapy	Improved physiological parameters	II

Level of evidence is categorized as follows: I – systematic reviews/meta-analyses; II – randomized or experimental studies; III – observational studies.

### Clinical perspective from practice (now referenced)

From a clinical standpoint, the integration of music into cardiovascular care represents a practical and accessible intervention, particularly in acute settings where rapid modulation of autonomic tone is desirable (1, 2). In emergency cardiology, patients frequently present with heightened sympathetic activation due to pain, anxiety, and hemodynamic instability (2, 13).

In such contexts, controlled auditory environments—especially the use of slow-tempo, rhythmically stable, and emotionally neutral or pleasant music—may contribute to stabilization of heart rate and subjective reduction of distress (1, 9, 15). While music cannot replace pharmacological therapy, it may serve as a complementary tool that enhances patient comfort and potentially improves physiological parameters (14, 15).

Importantly, the clinician's awareness of individual patient preferences appears crucial, as adverse or emotionally charged auditory stimuli may produce opposite effects (4, 11). The incorporation of music into clinical protocols should therefore be individualized and carefully monitored (4).

### Proposed clinical protocol for music-based modulation of heart rate in cardiovascular and emergency care

#### Rationale

Music has demonstrated consistent effects on autonomic regulation, particularly through modulation of sympathetic and parasympathetic activity. Previous studies have demonstrated that structured music interventions can reduce sympathetic activity and improve heart rate variability, supporting their role in autonomic regulation (1, 4, 9). These findings provide the physiological basis for the structured parameters proposed in this protocol. In acute cardiovascular conditions, excessive sympathetic activation contributes to increased heart rate, myocardial oxygen demand, and arrhythmogenic risk (2, 13).

A structured music-based intervention may therefore serve as an adjunctive strategy to support autonomic stabilization in both acute and chronic cardiovascular settings.

#### Patient selection

Eligible patients: acute myocardial infarction (hemodynamically stable), supraventricular arrhythmias (stable patients), patients in ICU with elevated stress and anxiety, postoperative cardiac patients, patients undergoing cardiac rehabilitation. Exclusion criteria: hemodynamic instability (shock, severe hypotension), severe cognitive impairment preventing auditory processing, agitation or intolerance to auditory stimuli, hearing impairment (relative contraindication).

#### Intervention parameters

Music characteristics (recommended): tempo: 60–80 BPM (the selection of this range is supported by studies demonstrating entrainment of physiological rhythms to external auditory stimuli, leading to improved autonomic balance and increased vagal activity) (1, 9), rhythm (regular, predictable), dynamics (low to moderate intensity), type (instrumental, ambient, or calming classical music), avoid (highly stimulating, loud, or emotionally distressing music), delivery method (headphones (preferred in ICU/emergency settings), controlled ambient audio (rehabilitation settings).

#### Session duration

Fifteen to thirty minutes per session, 1–3 sessions daily depending on clinical context. Session duration of 15–30 min is supported by previous studies demonstrating that this exposure time is sufficient to induce measurable autonomic changes while maintaining patient compliance (13, 15). This duration also reflects a balance between physiological efficacy and feasibility in clinical settings, particularly in intensive care and acute cardiovascular care environments.

#### Monitoring and outcomes

Primary parameters: heart rate (HR), blood pressure (BP), heart rate variability (HRV), if available. Secondary parameters. Anxiety scores (e.g., visual analog scale), respiratory rate, patient-reported comfort.

#### Expected physiological effects

Reduction in HR, increased parasympathetic activity (↑ HF component of HRV) (9, 12), reduction in perceived stress and anxiety (15, 17, 20–26).

#### Application in emergency cardiology

Acute myocardial infarction (AMI), apply after initial stabilization [goal: reduce sympathetic overactivation and myocardial oxygen demand (2, 13)], arrhythmias, use in stable supraventricular arrhythmias [goal: enhance vagal tone and rhythm stabilization (4, 9)], intensive care unit (ICU), apply during periods of agitation or stress [goal: reduce HR, anxiety, and environmental stress burden (14, 15)], emergency department, use in patients with anxiety, chest pain, or procedural stress [goal: rapid non-pharmacological reduction of sympathetic activation (15, 17)].

#### Safety considerations

Music intervention is generally safe and non-invasive. However: continuous monitoring is recommended in acute settings, immediate discontinuation if adverse response occurs (tachycardia, agitation), avoid overstimulation in critically ill patients.

#### Implementation considerations

Incorporate into existing clinical workflows, train staff for basic protocol application, use pre-selected validated playlists, consider patient preference when possible.

#### Future directions

Future studies should focus on large randomized trials, standardized protocols, and integration of AI-driven personalized interventions based on physiological monitoring (Table 2).

**Table 2:** Clinical protocol for music-based intervention in cardiovascular care.

Component	Description	Rationale	References
Patient Selection	Hemodynamically stable patients (AMI, arrhythmias, ICU stress, post-op, rehabilitation)	Ensures safety and applicability of intervention	Bradt et al. (13)
Contraindications	Hemodynamic instability, severe agitation, cognitive impairment	Avoids adverse responses to auditory stimuli	Nilsson (15)
Music Tempo	60–80 BPM	Synchronization with resting heart rate and promotion of parasympathetic activity	Bernardi et al. (1); Iwanaga et al. (9)
Music Type	Instrumental, ambient, calming classical	Reduces emotional overstimulation and variability in response	Trappe (10)
Delivery Method	Headphones (ICU/emergency) or ambient sound (rehabilitation)	Ensures controlled exposure and minimizes environmental noise	Bradt et al. (13)
Session Duration	15–30 min	Sufficient to induce autonomic changes while maintaining compliance	Nilsson (15); Bradt et al. (13)
Frequency	1–3 sessions daily	Balances therapeutic effect with clinical feasibility	Clinical practice-based recommendation
Monitoring	HR, BP, HRV, anxiety level	Evaluates physiological and psychological response	Mitrovic et al. (5)
Expected Effects	↓ HR, ↓ BP, ↑ HRV (vagal tone), ↓ anxiety	Reflects improved autonomic balance	Bernardi et al. (1)
Clinical Settings	ICU, emergency department, rehabilitation	Broad applicability across cardiovascular care	Bradt et al. (13)
Safety Measures	Continuous monitoring, discontinue if adverse response	Ensures patient safety	Nilsson (15)

Summary of recommended parameters for implementing music-based interventions, including patient selection, intervention characteristics, monitoring, and expected clinical effects. The protocol is based on current evidence and clinical feasibility considerations and should be considered exploratory pending validation in randomized controlled trials.

### Implementation algorithm (one-page clinical framework)

#### Overview

The implementation of music-based interventions in cardiovascular and emergency care requires a structured and reproducible approach. The following algorithm provides a simplified clinical framework for integrating music into routine practice.

#### Stepwise algorithm

Step 1: Patient Assessment, evaluate clinical status (stable vs. unstable), assess cognitive function and ability to perceive auditory stimuli, identify contraindications (agitation, intolerance to sound); Step 2: Indication, elevated heart rate due to stress/anxiety, acute myocardial infarction (post-stabilization), supraventricular arrhythmias (stable patients), ICU-related stress or sleep disturbance; Step 3: Music selection, tempo: 60–80 BPM, type: instrumental, ambient, or calming classical, avoid emotionally distressing or highly stimulating music, prefer patient-selected music when feasible; Step 4: Intervention delivery, use headphones (preferred in ICU/emergency settings), session duration: 15–30 min, frequency: 1–3 times daily; Step 5: Monitoring, heart rate (HR), blood pressure (BP), heart rate variability (HRV), if available, subjective anxiety level; Step 6: Evaluation of response, reduction in HR, improved HRV (↑ parasympathetic tone), decreased anxiety; Step 7: Adjustment, modify music type based on patient response, discontinue if adverse effects occur (agitation, tachycardia); Step 8: Integration, incorporate into standard care protocols, document response in clinical records, consider long-term use in rehabilitation.

### Proposed clinical study (randomized controlled trial design)

#### Study design

Prospective, randomized, controlled trial; Parallel-group design; Single-blind (outcome assessor blinded).

#### Study population

Inclusion criteria: adults ≥ 18 years, acute myocardial infarction (hemodynamically stable), or patients with supraventricular arrhythmias, or ICU patients with elevated stress/anxiety.

Exclusion Criteria: hemodynamic instability, severe cognitive impairment, hearing impairment, severe agitation or psychiatric instability.

#### Randomization

1:1 allocation, intervention group vs. control group.

#### Intervention

Intervention group: music session (60–80 BPM, calming), 20 min, twice daily, headphones. Control group: standard care (no music intervention), Optional: neutral auditory condition (silence or white noise).

#### Outcomes

Primary endpoint: change in heart rate (ΔHR). Secondary endpoints: HRV parameters (HF, LF/HF ratio), blood pressure, anxiety scores, arrhythmia incidence (if applicable).

#### Data collection

Baseline measurement, during intervention, post-intervention (immediate and 24 h follow-up).

#### Statistical analysis

Continuous variables: *t*-test or *Mann–Whitney U*, Repeated measures: ANOVA or mixed models, significance level: *p* < 0.05.

#### Sample size (proposal)

Estimated: 80–120 patients, based on expected moderate effect size in HR reduction.

#### Expected results

Reduction in HR in intervention group, improved HRV (↑ parasympathetic activity), reduced anxiety.

#### Clinical impact

If confirmed, this intervention could: provide a low-cost adjunct therapy, improve autonomic stability in acute settings, reduce need for sedatives or anxiolytics.

## Conclusion

Music exerts a measurable influence on heart rate and autonomic regulation through complex neurophysiological mechanisms. Its integration into cardiovascular care, including emergency settings, represents a promising non-pharmacological approach. Further research is required to standardize its application and confirm clinical benefits.
